# Development and dosimetric verification of **3**D customized bolus in head and neck radiotherapy

**DOI:** 10.1093/jrr/rrac013

**Published:** 2022-04-14

**Authors:** Nichakan Chatchumnan, Sakda Kingkaew, Chuanchom Aumnate, Taweap Sanghangthum

**Affiliations:** Division of Radiation Oncology, Department of Radiology, King Chulalongkorn Memorial Hospital, Bangkok 10330, Thailand; Division of Radiation Oncology, Department of Radiology, King Chulalongkorn Memorial Hospital, Bangkok 10330, Thailand; Metallurgy and Materials Science Research Institute Chulalongkorn University, Bangkok 10330, Thailand; Division of Radiation Oncology, Department of Radiology, Faculty of Medicine, Chulalongkorn University, Bangkok 10330, Thailand

**Keywords:** silicone rubber, flat bolus, 3D customize bolus, 3D printer

## Abstract

The commercial flat bolus cannot form perfect contact with the irregular surface of the patient’s skin, resulting in an air gap. The purpose of this study was to evaluate the feasibility of using a 3D customized bolus from silicone rubber. The silicone rubber boluses were studied in basic characteristics. The 3D customized bolus was fabricated at the nose, cheek and neck regions. The point dose and planar dose differences were evaluated by comparing with virtual bolus. The hardness, thickness, density, Hounsfield unit (HU) and dose attenuation of the customized bolus were quite similar to a commercial bolus. When a 3D customized bolus was placed on the RANDO phantom, it can significantly increase buildup region doses and perfectly fit against the irregular surface shape. The average point dose differences of 3D customized bolus were −1.1%, while the commercial bolus plans showed −1.7%. The average gamma results for planar dose differences comparison of 3D customized bolus were 93.9%, while the commercial bolus plans were reduced to 91.9%. Overall, A silicone rubber bolus produced the feasible dosimetric properties and could save cost compared to a commercial bolus. The 3D printed customized bolus is a good buildup material and could potentially replace and improve treatment efficiency.

## INTRODUCTION

Radiotherapy is one of the most common methods used for the treatment of cancer patients. In order to deliver a sufficient radiation dose to the tumor, adequate types of radiation are selected depending on the tumor location. Conventionally, the electron is applied to treat superficial lesions such as skin cancer, while the high-energy photon is used to treat deeply located lesions. However, a sufficient dose cannot be delivered to the surface with the high-energy photon treatment due to skin sparing effect. To avoid this limitation, several types of commercially available boluses are often used [1]. These bolus materials should be nearly tissue equivalent and allow a sufficient surface dose enhancement. In practice, most commonly used commercial flat boluses cannot form perfect contact with the irregular surface of the patient’s skin, particularly the nose, ear and scalp, resulting in the air gap effects with second skin sparing effect and reduces both the maximum and surface doses [2–6]. Thus, commercial flat boluses need to be used with great care, especially when the skin has a particularly irregular shape. To overcome this drawback of the commercially flat bolus, 3D printing technology advances significantly benefit from producing customized shapes [7, 8].

The 3D printing skills can be applied to create individually customized boluses, which were designed to compensate for the irregular surface in radiotherapy. In general, two ways have been reported of making a bolus. The first method is directly printed a bolus with 3D printing materials after the design stage. Polylactic acid (PLA) is commonly used as a printing material, which had been demonstrated by Burleson *et al.* [9]. Moreover, Park *et al.* [10] and Ricotti *et al.* [11] reported that the doses of 3D printed PLA bolus in phantom simulating radiotherapy of breast cancer after radical resection were more uniform than with the commercial bolus. Acrylonitrile butadiene styrene (ABS) copolymer is another printing material generally used. Both PLA and ABS are suitably used as direct printing materials; however, both are too hard and uncomfortable for patients. The second method is to print the shells of the bolus and then fill them with other soft materials to create a customized and soft bolus. Richard *et al.* [12] printed the shell in PLA using the 3D printer and filled it with silicone rubber for non-melanoma skin cancer electron beam radiotherapy. Silicone rubber has advantages when making a bolus due to its excellent biocompatibility, chemical stability and good mechanical properties. In their study, the customized boluses were fabricated with 3D printing technology to create the bolus shells that can be perfectly fitted to the target irregular shape, nose, cheek and neck regions. Two kinds of silicone rubbers were used as soft materials to fill in the 3D printed customized shells, resulting in the customized boluses. The dosimetric properties and physical properties of the fabricated customized boluses were then evaluated comparatively concerning a commercial flat bolus to investigate the feasibility of overcoming the drawbacks of currently used commercial flat boluses.

## MATERIALS AND METHODS

### Bolus fabrication

**Fig. 1.**
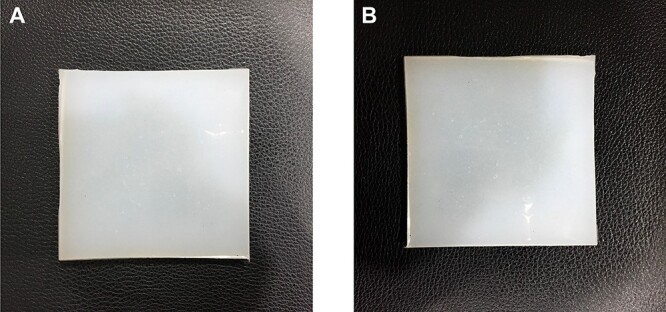
The silicone rubber bolus of: (a) RA-00AB; (b) RA-05AB.

Two kinds of silicone rubbers, RA-00AB and RA-05AB, were selected as the recommendation from the company. The former is based on vinyl silicone oil and white carbon black, while the latter one is based on Hydroxy-terminated Poly-dimenthylsiloxane, Aqua and Titanium Dioxide. Each model consists of two solution bottles, A and B. The solutions in bottles A and B were carefully mixed to avoid bubbles at room temperature with a ratio of 1:1 and filled in the container with a size of 10 × 10 × 1 cm^3^. The recommended hardening time of the mixed solution was 6–8 hours, however, we waited 24 hours before using it. The RA-00AB and RA-05AB boluses are presented as shown in Figs 1a and 1b, respectively.

### Physical properties verification

The 1 cm thick in-house boluses, RA-00AB and RA-05AB, with the size of 10 × 10 cm^2^ were performed using a CT scan by Siemens SOMATOM Definition AS scanner (Global Siemens Healthcare, Erlangen, Germany) and the images were exported to Eclipse treatment planning system (TPS) (Varian Medical Systems, Palo Alto, CA). The 6 MV beams and 10 × 10 cm^2^ field at source to surface distance were planned and evaluated in dosimetric from point dose differences at 0.5, 1.0 and 1.5 cm depths and planar dose differences at 1.5 cm depth by comparing with virtual bolus created in TPS using 3%/2 mm gamma criteria from SNC patient software (Sun Nuclear Corporation, Melbourne, FL). The dose attenuation effect was studied, and the hardness of boluses was evaluated using Shore A Durometer, which is equipment for measuring the hardness of different materials. Shore A hardness scale measures the hardness of flexible rubber, silicone, vinyl and soft plastics that range from very soft and flexible to hard with almost no flexibility with 0–100 shore A hardness scales or HA.

**Fig. 2.**
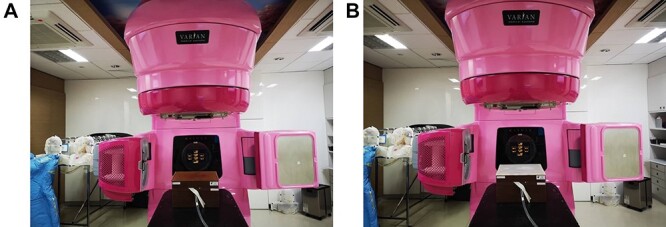
The dose attenuation measurement setting up for: (a) commercial bolus; (b) customized bolus.

From the dosimetric and hardness evaluation, the vinyl silicone-based rubber bolus (RA-00AB model) presented better dosimetric and hardness results. Thus, it was then chosen for further study of the basic physics properties. In this section, the RA-00AB was used to fabricate a flat bolus with the size of 30 × 30 × 1 cm^3^. The bolus was scanned by a CT simulator and exported to TPS. The obtained bolus characteristics, such as hardness, thickness, density, Hounsfield unit (HU) and dose attenuation, were also investigated and compared to commercial flat bolus, Bolx-I (CIVCO Medical Solution, Orange City, FL) [13]. The thicknesses were measured with a vernier caliper. The density was calculated using the density formula as the total weight of the bolus per unit volume. The HU of the bolus was measured for 9 positions on TPS. The dose attenuation verification part was measured at 5 cm depth, 10 × 10 cm^2^ by FG65-P detector (Scanditronix-Wellhofer, IBA, Uppsala, Sweden) in the solid phantom (Gammex, Middleton, WI) as presented in Fig. 2. Moreover, the characteristics between irradiated and non-irradiated boluses were compared by attenuated total reflection Fourier transform infrared spectroscopy (ATR-FTIR) to determine the changes in chemical functional groups of the RA-00AB bolus after irradiation. Furthermore, the thermal stability of such bolus after 70 Gy irradiation was characterized using thermogravimetric analysis (TGA) technique.

**Fig. 3.**
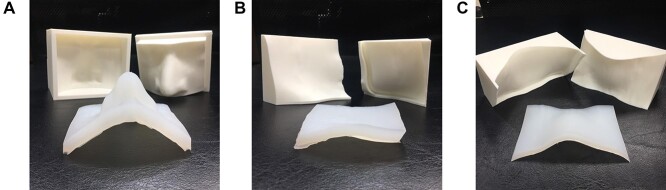
The bolus shells printed by 3D printer and 1 cm 3D customized bolus for: (a) nose; (b) cheek; (c) neck regions.

The three bolus shells were designed to create boluses at the nose, cheek and neck regions using the Autodesk Fusion 360 program, which took about 6–8 hours per piece, depending on the experience. The obtained STL files (3D bolus shell) were then sliced into layers using a CreatWare program and then printed out by the 3D printer, create bot D600. The printout process used 15–20 hours per piece. The 1 cm silicone rubber solutions were filled to the shells to fabricate the customized 3D printed boluses as presented in Fig. 3. The Gafchromic EBT3 films (Ashland ISP, Wayne, NJ) were inserted between slabs of the Alderson RANDO phantom (Radiology Support Devices Inc., Long Beach, CA). The customized 3D printed boluses and commercial bolus were placed on the RANDO phantom in the specified areas. The 6 MV, 10 × 10 cm^2^ field perpendicular to phantom surface plans in each area were calculated on TPS and irradiated by a treatment machine.

The various depths of point dose differences along the central axis on Gafchromic EBT3 films were measured compared to the calculated dose with virtual bolus in TPS. The dose from TPS with virtual bolus was used as the reference because the virtual bolus can make the perfect match according to the curvature of the phantom.

For planar plane analysis, the SNC patient software was selected to evaluate the calculated dose differences between a treatment plan with commercial/customized boluses and a treatment plan with a virtual bolus at the central axis plane. The gamma index with 3%/2 mm gamma criteria and 10% threshold was used for analysis.

## RESULTS

### Physical evaluation

The RA-00AB model bolus was softer than the RA-05AB model. The measured point dose in all depths of both RA-00AB model and RA-05AB model boluses showed less than 0.3% deviation from the calculated dose under the virtual bolus condition, as presented in Table 1. Moreover, the planar dose differences showed a perfect agreement in both types of boluses with 100% gamma passing rate results. The dosimetric property of both types of silicone rubber bolus was quite similar. However, RA-00AB was significantly softer than RA-05AB with the hardness values of 1.5 HA and 5.0 HA for RA-00AB and RA-05AB, respectively. Thus, the RA-00AB silicone rubber bolus model was selected as a potential bolus for further study in the next step.

**Table 1:** The point dose differences between virtual bolus in TPS and in-house boluses of RA-00AB and RA-05AB

**Depth (cm)**	**Virtual Bolus Dose (cGy)**	**RA-00AB**		**RA-05AB**	
		**Measured Dose (cGy)**	**Dose Diff. (%)**	**Measured Dose (cGy)**	**Dose Diff. (%)**
0.5	209.3	209.8	0.24	209.8	0.24
1.0	206.7	206.1	−0.29	206.5	−0.10
1.5	202.3	201.7	−0.30	201.9	−0.20

The physical properties of 30 × 30 × 1 cm^3^ customized RA-00AB bolus were compared with commercial super flab bolus. The average thickness of commercial bolus was 1.05 ± 0.00 cm and customized bolus was 1.07 ± 0.01 cm, while density was 1.03 and 0.99 g/cm^3^, respectively. The HU of commercial bolus and customized bolus were −124 ± 63 and −73 ± 43, respectively. There were good agreements between the dose attenuation of commercial bolus and customized bolus at 5 cm within solid water phantom, that were 167.2 and 167.6 cGy, respectively. The point dose differences were only 0.2%.

**Fig. 4.**
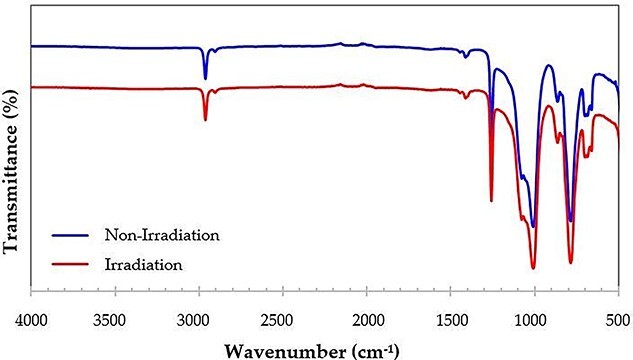
ATR-FTIR spectra of non-irradiation bolus and irradiation bolus (note: both non-irradiation and irradiation curves are superimposed but the curve of irradiation is shifted down to illustrate the same pattern of both spectrum).

In general, the spectrum obtained from the ATR-FTIR analysis can represent a molecular fingerprint of the sample. In this study, the ATR-FTIR test was used to observe the changes in chemical functional groups of the bolus after 70 Gy irradiation. The results showed the overlapping spectra between irradiation bolus and non-irradiation bolus, as shown in Fig. 4, indicating that the molecular functional group of the bolus remained unchanged after the irradiation process.

**Fig. 5.**
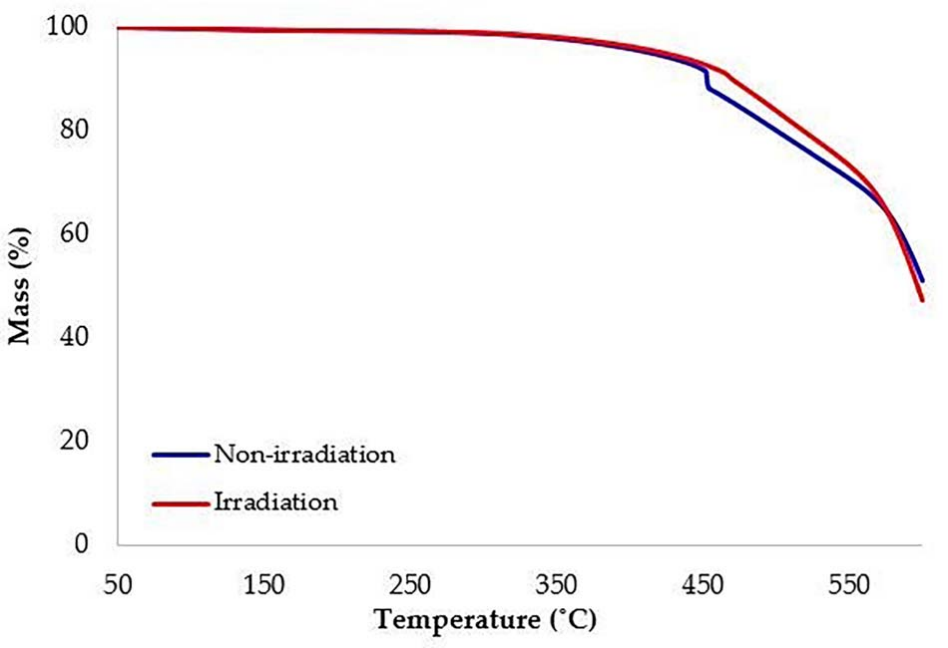
Thermogravimetric curves of non-irradiation and irradiation boluses.

**Fig. 6.**
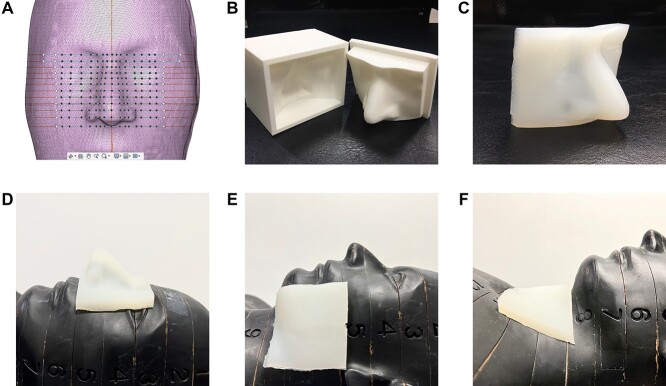
The procedure of making silicone rubber bolus based on 3D printing technology at nose area from: (a) Fusion 360 program for bolus shell designed; (b) bolus shell fabricated by 3D printer; (c) silicone rubber bolus; (d) bolus on nose; (e), bolus on cheek; (f) bolus on neck.

Moreover, the TGA analysis was used to evaluate the thermal stability of the bolus after irradiation. Figure 6 presented the mass loss as a function of temperature. At around 450°C, the non-irradiation bolus showed a stepwise decrease in mass, which may be due to the vinyl silicone residue from incomplete crosslinking of RA-00AB during the forming process as shown in Fig. 5. After irradiation, this stepwise decrease was not observed. The radiation process may enhance the crosslinking of RA-00AB, leading to more thermal stability of the irradiation bolus. The degradation temperatures of the non-irradiation bolus and irradiation bolus were 452.5°C and 469.1°C, respectively. These findings indicate that irradiation has a slight effect on improving the thermal stability of the bolus. Moreover, the high degradation temperature in a range of 450–500°C was ensuring that the boluses were not affected by the temperature in clinical use.

### Clinical application

The 3D slicer and Fusion360 program were used to design the bolus shells, and the 3D printing technology was used to print the shells. The shells were filled with silicone rubber RA-00AB as shown in Fig. 6.

**Fig. 7.**
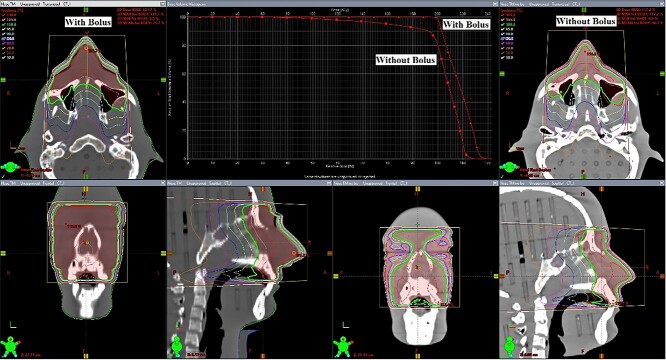
Dose distribution and DVH comparison between with and without bolus.

Figure 7 shows the dose distribution and dose volume histogram comparison between the plan with and without bolus of simple anterior-to-posterior field. It was very clear that in the coronal plane at 5 cm depth, the plan with bolus could significantly increase the surface and buildup region doses and be more suitable to apply in superficial tumor cases. Table 2 shows the average point dose differences from EBT3 film using 3D customized/commercial boluses to calculated doses with virtual bolus in the nose, cheek and neck area. On the average, 3D customized bolus presented the slightly lower dose differences than commercial bolus results.

The SNC patient software was used to compare planar dose differences. The gamma pass rate of 3D customized bolus for nose, cheek and neck regions were 90.0%, 92.4% and 99.4% gamma passing rate, respectively. For the planar dose difference of commercials bolus, the gamma pass rate for nose, cheek and neck regions were 88.1%, 91.7% and 95.8%, respectively.

## DISCUSSION

With the bolus, the skin dose was significantly increased, agreeing with the results from the study of Shin *et al.* [14]. They fabricated a customized 3D bolus using a 3D printer and evaluated its feasibility in clinical practice by comparing its performance without a bolus in the TPS. The results indicated that the 3D printed customized bolus is a good buildup material for cancer treatment planning.

Between the two models of silicone rubbers, the gamma passing rate at 3%/2 mm gamma criteria also presented the excellent agreement with 100% pass rate in both types of bolus, and the point dose differences were also not much different. However, the RA-00AB model was softer than the RA-05AB model. It could be implied that the RA-00AB model bolus should make more contact with the surface than another model. When the size of 30 × 30 × 1 cm^3^ was fabricated, the physical characteristics were quite the same as a commercial bolus for all hardness, thickness, density, HU and dose attenuation properties that means the RA-00AB silicone rubber can be replaced with the commercial super flab bolus at a lower price.

When the commercial super flab bolus with the standard size of 30 × 30 × 1 cm^3^ was placed to the irregular shape of the phantom, especially in the head and neck area, a lot of tapes were used to reduce the air gap between bolus and phantom. It was not convenient in real patient cases, had difficult reproducibility and still presented a large air gap. This limitation can be solved with a 3D customized bolus using 3D printing technology. The 3D printed bolus was very good fit against the irregular surface of the rando phantom that can significantly reduce the air gap between bolus and phantom compared with commercial bolus.

In the clinical situation, the average point dose differences in all regions were within the IAEA TRS-430 recommendation [15]. The largest dose differences from the virtual bolus were observed in the cheek region due to the difficulty of defining the exact position on the slope tissue. However, the lowest gamma pass rate was found in the nose region on the planar plane in TPS because this area includes inhomogeneity of the large air cavities at the nasal. The 3D customized bolus plans showed a higher gamma pass rate in all regions than those of the commercial flat bolus, especially in the buildup region that commercial bolus presented the large air gap, contributing to re-buildup dose after air gap.

As well as the photon dose application, this silicone-based bolus is also a good option to apply for electron beam treatment. The silicone-based bolus could be used to compensate for the irregular surface and increase the surface dose in case of superficial tumor treatment. In many clinical situations, the commercial boluses were cut into the shape of a treatment field that is very high cost. This silicone bolus is a cost-effective option.

**Table 2:** The average point dose differences between measurement (3D customized bolus and commercial bolus) and calculation (virtual bolus)

**Regions**	**Average point dose differences (%)**		***P*-value**
	**3D customized bolus**	**commercial bolus**	
Nose	−0.19 ± 2.77	−0.24 ± 2.76	0.783
Cheek	−3.48 ± 4.21	−4.65 ± 4.16	0.001
Neck	0.28 ± 1.80	−0.09 ± 2.18	0.016

## CONCLUSION

A silicone rubber bolus produced the feasible physical and dosimetric properties of a commercial bolus and could be 15 times save cost when compared to a commercial bolus. The 3D printed customized bolus is a good buildup material, high reproducibility of daily setup and potentially to replace commercially flat bolus.

## PRESENTATION AT A CONFERENCE

Oral presentation at the 20th Asia-Oceania Congress on Medical Physics (AOCMP)—18th South-East Asia Congress of medical Physics (SEACOMP)—12th Annual Meeting of Thai Medical Physicist Society (TMPS), Phuket, Thailand, 2020.
